# The Antioxidant Effect of *Medicago sativa* L. (Alfalfa) Ethanolic Extract against Mercury Chloride (HgCl_2_) Toxicity in Rat Liver and Kidney: An In Vitro and In Vivo Study

**DOI:** 10.1155/2021/8388002

**Published:** 2021-08-25

**Authors:** M. Raeeszadeh, M. Moradi, P. Ayar, Abolfazl Akbari

**Affiliations:** ^1^Department of Basic Sciences, Sanandaj Branch, Islamic Azad University, Sanandaj, Iran; ^2^Graduate of Faculty of Veterinary Sciences, Sanandaj Branch, Islamic Azad University, Sanandaj, Iran; ^3^Department of Physiology, School of Veterinary Medicine, Shiraz University, Shiraz, Iran

## Abstract

Heavy metals such as mercury are some of the environmental pollutants and can induce toxicity by bioaccumulation and oxidative damage. This study aimed to investigate the effect of ethanolic extract of *Medicago sativa* L. (Alfalfa) on mercury damage in the kidney and liver of rats. Thirty Wistar rats were randomly divided into five groups, the control group, S group (2 mg/kg mercury chloride), and T1, T2, and T3 groups that, in addition to mercury, received doses of 250, 500, and 750 mg/kg of the alfalfa extract. On the last day, blood samples were taken, and the serum was separated to measure biochemical and oxidative stress parameters in the kidney and liver. A part of the kidney and liver was also used for histopathological evaluation. Total phenols and flavonoids were 40.45 ± 2.12 and 14.36 ± 0.45 mg/g, respectively, whereas IC_50_ was 245.18 ± 19.76 *μ*g/ml. The body weight significantly decreased in the S group compared to other groups, while treatment with different doses of alfalfa extract increased the body weight. Mercury concentration in the kidney was higher than that in the liver. The serum levels of urea, creatinine, alanine aminotransferase (ALT), and alkaline phosphatase (ALP) significantly increased in the S group compared to the control group, while treatment with different doses of alfalfa extract increased their levels. Moreover, an increase in malondialdehyde (MDA) and a decrease in glutathione peroxidase (GPx), catalase (CAT), total antioxidant capacity (TAC), and superoxide dismutase (SOD) activity were observed in the S group. The level of these parameters significantly improved in the groups receiving the extract compared to the S group. Furthermore, the histopathological evaluation showed glomerular and tubular damage and hepatic necrosis in the S group and that these conditions improved in the T3 group. The findings of this study showed that the ethanolic extract of alfalfa in a dose-dependent manner has potentially unique protective effects against mercury poisoning in the kidney and liver.

## 1. Introduction

Mercury is a heavy metal with a dual capacity and has no biological function in the body. Exposure to it causes damage to growing and mature organisms. Mercury exposure, first, affects the central nervous system and then the kidneys and the digestive system [1]. The biological, pharmacokinetic, and clinical symptoms of mercury poisoning vary according to its chemical structure and duration of its exposure. Mercury vapors have a strong affinity for sulfhydryl groups when they enter the body and bind to sulfur-containing amino acids. Mercury dissolves in serum, attaches to the membrane of red blood cells, and is transmitted to the brain. Mineral mercury crosses the placenta and the blood-brain barrier and easily accumulates in the fetal brain [2]. In addition to the brain, mercury can accumulate in the thyroid, myocardium, muscles, adrenal glands, liver, kidneys, sweat glands, pancreas, enterocytes, salivary glands, testes, and prostate whereas impairs the function of these organs [3, 4]. Mercury has a strong tendency to revive sulfur and especially thiol-like molecules such as glutathione (GSH), cysteine, and metallothionein. The toxic effects of mercury on organs such as the liver and kidneys are due to biological reactions with metallothionein, glutathione, and albumin [5]. Many studies showed that oxidative stress caused by mercury exposure (organic and inorganic) results from a reduction in thiol-containing groups, including glutathione. Mercury has been reported to reduce glutathione and induce lipid peroxidase and increase hydrogen peroxide formation in rat kidney tissue [6]. Several studies showed that many plants are considered rich sources of antioxidants and can be useful in preventing or treating diseases caused by an imbalance of the oxidative-antioxidative system. In consideration of the fact that oxidative stress induced by exposure to environmental pollutants such as mercury may lead to kidney and liver poisoning, researchers believe that antioxidant compounds can play a protective role against these pathological conditions [7]. Alfalfa, *Medicago sativa* L., is one of the most famous traditional medicinal plants used to cure and prevent many diseases [8]. Alfalfa contains vitamins (A, B1, B6, B12, C, D, E, and K), amino acids, sugars, proteins, minerals (Fe, Zn, Cu, Al, B, Cr, Co, Mn, Mo, Se, Si, Na, Ca, P, K, and Mg), and other nutrients. Due to its richness in vitamins and phytoestrogens, this plant is used as a food additive in several countries [8]. Phytochemical studies indicated that this plant contains a variety of secondary metabolites including flavonoids, alkaloids, phytoestrogens (coumestrol, daidzein, genistein, and pyocyanin), and coumarins as potent antioxidants [9, 10]. Now, the question is whether the consumption of this plant extract can prevent kidney and liver damage caused by exposure to mercury? Moreover, due to the lack of national and international studies as well as the importance of the toxicity of heavy metals, the subject of this study was to investigate the effect of hydroalcoholic extract of alfalfa on kidney and liver damage resulted from exposure to mercury chloride in male rats.

## 2. Materials and Methods

### 2.1. Preparation of the Alfalfa (*Medicago sativa*) Extract

After collection from the farmstead of Sanandaj, Iran, alfalfa plants were stored in a dry place and kept away from sunlight to dry, and then the leaves of the plant were pulverized. After that, 150 g of crushed powder with 75% ethanol were reached a volume of one liter and was immersed for 48 hours. The mixture was filtered by Whatman No. 1 filter paper and condensed by a rotary at 40°C. Finally, in order to administer the dose to the animals, concentrations of 250, 500, and 750 mg/kg of the extract were prepared by distilled water [9].

### 2.2. Measurement of Antioxidant Activity of the Ethanolic Extract

The measurement of antioxidant activity of the ethanolic extract was performed based on the inhibition of 2,2-diphenyl-1-picrylhydrazyl (DPPH) by extract. The concentrations of 20, 50, 100, 200, 500, 700, and 1000 *μ*g/ml of the extract were prepared; after that, 300 *μ*l extract was added to 2.7 ml of DPPH reagent and then read at 520 nm wavelength. IC_50_ was determined as the concentration of the extract that could inhibit 50% of DPPH radicals. This procedure was performed against vitamin C [11]. Total phenol content was measured using the Folin-Ciocalteu method. This low-cost method is the most widely used method for measuring polyphenols in plant extracts.

It is worth noting that the gallic acid was used to draw the calibration curve. The obtained results were then expressed as gallic acid equivalents (GAE) in mg/g of the extract [12].

The total flavonoid content of the extract was measured using aluminum chloride by the colorimetric method [13].

### 2.3. Study Design

The present study was experimental-interventional. Thirty adult male Wistar rats weighing 200 to 220 g and 6–8 weeks old were purchased from Pasteur Institute of Iran (Tehran). Animals were kept under controlled environmental conditions (18–20°C, 12 hours of light, and 12 hours of darkness) and had free access to water and chow during the study. This study was started 7 days after the adaptation of animals to environmental conditions. This study was approved by the Ethics Committee of Kurdistan University of Medical Sciences (IR.MUK.REC.1397/5004). To induce experimental poisoning, mercury chloride was administered orally at a dose of 2 mg/kg body weight daily for 30 days [14]. Rats were randomly divided into five groups:

  C group: healthy animals received only distilled water
  S group: the animals received mercury chloride (2 mg/kg/day) orally
  T1 group: the animals received mercury chloride (2 mg/kg) + alfalfa ethanolic extract (250 mg/kg) intraperitoneally daily
  T2 group: the animals received mercury chloride (2 mg/kg) + alfalfa ethanolic extract (500 mg/kg) intraperitoneally daily
  T3 group: the animals received mercury chloride (2 mg/kg) + alfalfa extract (750 mg/kg) intraperitoneally daily

The duration of the study was 30 days. The basal and final body weights of the animals were measured. The doses of alfalfa ethanolic extract were selected according to earlier studies [15–17].

### 2.4. Sampling, Preparation, and Measurement of Biochemical Parameters

At the end of the study, the experimental groups were anesthetized by intraperitoneal injection of ketamine (100 mg/kg) and xylazine (10 mg/kg) [18]. Blood sampling was taken from the animal's heart; then samples were centrifuged and the serum was separated to measure the amount of total antioxidant capacity (TAC) by using Ferric Reducing Antioxidant Power (FRAP). Urea, creatinine, uric acid, sodium, and potassium were measured as the parameters of renal biochemistry and Alanine Aminotransferase (ALT), Aspartate transaminase (AST), and Alkaline phosphatase (ALP) concentration to assess for liver damage by standard commercial kits (Pars Test) by an autoanalyzer device (Hitachi, Japan). Further, malondialdehyde (MDA), glutathione peroxidase (GPx), catalase (CAT), and superoxide dismutase (SOD) concentration were evaluated by ZELLBIO kit, Germany. In addition, the concentration of mercury in the kidneys and liver was measured by the atomic absorption method.

### 2.5. Measurement of the TAC Level Using the FRAP Method

TAC values were measured by using a standard concentration chart of 100–1000 *μ*mol/l. In this method, the plasma's ability to regenerate the ferric iron is measured. At acidic pH, when the Fe III-TPTZ complex is regenerated to Fe II, it produces a blue color that reads at 593 wavelengths [19].

### 2.6. Measurement of GPx, CAT, and SOD Activity in the Liver and Kidney Tissues

After washing the liver and kidney with ice normal saline and placing it inside the microtube, the tissues were frozen in liquid nitrogen at a temperature of −80°C. At the time of the experiment, liver and kidney tissues were homogenized with a homogenizer and the supernatant obtained was used for biochemical testing. Catalase activity was measured by the Aebi method based on H_2_O_2_ decomposition at 240 nm [20]. Glutathione peroxidase (GSH-Px) catalyzes the oxidation of glutathione by the cumene hydroperoxide. The oxidized glutathione is then converted back to regenerative glutathione in the presence of the enzyme's glutathione reductase and nicotinamide adenine dinucleotide phosphate (NADPH). This reaction was read at 340 nm. The basis of measurement superoxide dismutase (SOD) activity was to inhibit the conversion of superoxide to H_2_O_2_ and O_2_ at 505 nm [21].

### 2.7. Measurement of the MDA Level in the Liver and Kidney Tissues

Trichloroacetic acid (TBA) was added to homogenized tissue. After precipitation of proteins, TBA was added to the top solution and it was boiled in bain-marie for 30 min, the absorption of light at 532 nm wavelength was read and calculated by the standard diagram. Its level was reported as *μ*g/g [22].

### 2.8. Measurement of Mercury in the Liver and Kidney Tissues by the Atomic Absorption Method

After drying the samples in an oven at 60°C, they were prepared by the wet digestion method. 0.5 g of kidney and liver tissues, 25 ml of concentrated sulfuric acid, 20 ml of 7 molar nitric acids, and 1 ml of 2% sodium molybdate solution were added. Moreover, a few welded stones were added to heat evenly. After cooling, from the top of the tube, 20 ml of nitric acid and concentrated perchloric acid was added in a ratio of 1 to 1. Then, it was heated until the white acid vapor disappeared. After cooling, 10 ml of water was added and it was heated for 100 minutes to obtain a uniform and clear solution. Finally, with standard diagram (HNO3 10% (V/V) + HCL 7% (V/V) + NaCl 0.02% (W/V)), concentrations based on the *μ*gr/gr weight unit were read [23].

### 2.9. Histopathological Examination of the Liver and Kidney

Histopathological examination was performed by fixing kidney and liver tissues in 10% formaldehyde solution. Then, sections of 5 *μ*m in diameter were prepared and stained with hematoxylin and eosin. The evaluation was performed with a light microscope and 40 magnification [24].

### 2.10. Statistical Methods

The study data were reported as mean ± SD. To compare the average parametric data among the groups, one-way ANOVA and Tukey's test were utilized. Liver and kidney histopathological scores were evaluated by the Kruskal–Wallis test.

## 3. Results

The results of the percentage of DPPH inhibition by extract and vitamin C are presented in Table 1. The results showed that the percentage of DPPH inhibition by the extract was in a dose-dependent manner. The percentage of DPPH inhibition from concentrations of 20 to 1000 *μ*g/ml was 15.78 ± 1.10 to 68.24 ± 3.33, respectively, and for vitamin C, it increased from 27.45 ± 4.63 to 84.12 ± 2.35. IC_50_ of the extract was 245.18 ± 48.41 *μ*g/ml. The total phenolic and flavonoid contents in Alfalfa ethanolic extract were 40.45 ± 5.19 (mg/g) and 14.36 ± 1.10 (mg/g), respectively (Table 1).

At the end of the study, the body weight decreased in the S group compared to the initial weight with other groups. There was a significant difference between the body weight in the control group and other groups (*P* < 0.001) (Table 2).

### 3.1. Serum Level of TAC in the Control and Treated Groups

**Figure 1:**
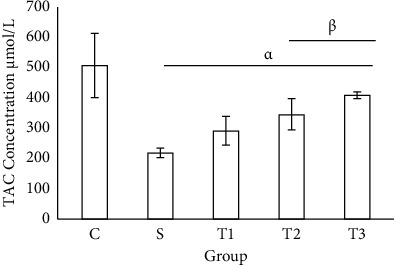
Evaluation of TAC concentration in serum of animals in different groups. Each column represents the mean ± standard deviation (SD). *P* < 0.001. *α* and *β* denote comparison with C and S groups.

The lowest concentration of TAC was 220 ± 15.49 *μ*mol/L in the S group, and the highest concentration was 508.65 ± 105.31 *μ*mol/L in the control group. There was a significant difference between the control group and other groups. The concentration of TAC significantly decreased in the S group compared with T2 and T3 groups (Figure 1).

### 3.2. GPx, CAT, SOD, and MDA Levels in the Liver Tissue of the Control and Treated Groups

**Figure 2:**
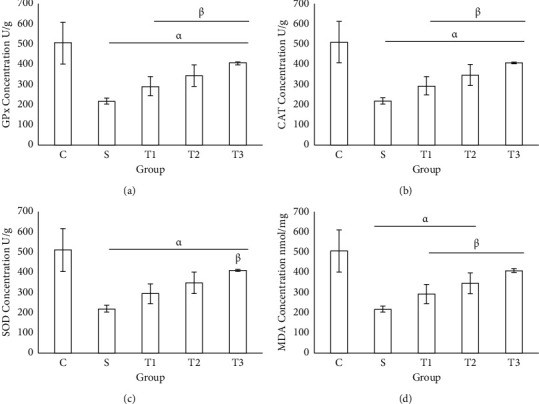
Determination of oxidative stress biomarker in the liver tissue of animal studied. Each column represents the mean ± standard deviation (mean  ±  SD). *P* < 0.001; *α* and *β* denote comparison with C and S groups.

The GPx activity in liver tissue of the S group significantly decreased compared to the control group (*P* < 0.001, Figure 2). The GPx activity significantly decreased in the S group compared to other groups (Figure 2(a)). The lowest activity of CAT was in the S group (25.9 ± 3.20) and the highest was in the control group (55.8 ± 2.04) while its activity insignificantly increased in the groups receiving the extract (Figure 2(b)). The SOD activity in the S group had a significant difference compared with other groups (*P* < 0.001) (Figure 2(c)), while its activity in T1, T2, and T3 improved compared to the S group. MDA concentration in the S group had a significant difference compared to other groups, while treatment with different doses of the extract could improve its level (Figure 2(d)).

### 3.3. GPx, CAT, SOD, and MDA Concentration in the Kidney Tissue of the Control and Treated Groups

**Figure 3:**
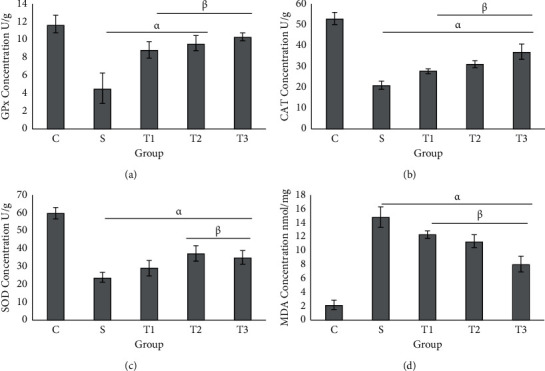
Determination of oxidative stress markers in the kidney tissue of animals in different groups. Each column represents the mean ± standard deviation (SD). *α* and *β* denote comparison with C and S groups.

The highest activity of GPx in kidney tissue was observed in the control group. The level of this enzyme in the S group had a significant difference with T1 and T2 groups (Figure 3(a)). The highest levels of CAT and SOD were observed in the control group at which this level had a significant difference compared with other experimental groups (Figures 3(b) and 3(c)). The lowest concentration of MDA in kidney tissue was in the control group and the highest level was in the S group (Figure 3(d)). There was a significant difference between the control and the S groups compared with other experimental groups (Figure 3(d)).

### 3.4. Serum Levels of Liver Enzymes and Hepatic Mercury Content in the Control and Treated Groups

The serum levels of AST, ALT, and ALP significantly increased in the S group compared to other groups. The level of ALT and ALP decreased in the T3 group compared to the S group. The content of mercury significantly increased in all groups compared to the control group (Table 3).

**Figure 4:**
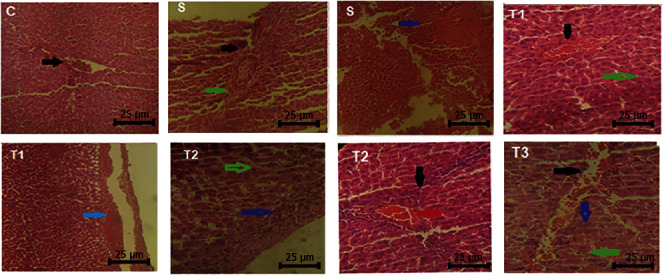
Histopathological changes in the liver of animals in the control and treated groups (H&E staining, 40x), scale bar = 25 *μ*m. C represents a normal structure of healthy hepatocytes and shows no evidence of degeneration. The nucleolus is euchromatin and active. The central veins and port triads are clear (black arrow) and have a normal structure. There is no infiltration of inflammatory cells into the parenchyma, and there is no bleeding or hypertension in the liver parenchyma. S represents necrosis of liver cells, pycnotic and hyperchromic nuclear cells (green arrow), deposition of cytoplasmic proteins and eosinophilization (blue arrow), parenchymal hemorrhage, severe infiltration of mononuclear inflammatory cells into the area around the portal vein, and also the presence of fibroblasts in the preport area (black arrow). T1 represents infiltration of mononuclear inflammatory cells in the peripheral area (black arrow), central vein congestion (red arrow), acidophilic cytoplasm of hepatocellular (green arrow), and hemorrhage under the Glisson's capsule (blue arrow). T2 represents infiltration of mononuclear inflammatory cells of lymphocytes and plasma cells around the triad port (black arrow), hyperplasia of Kupffer cells, the normal nucleus of hepatocytes (green arrow), and presence of mononuclear inflammatory cells on Glisson's capsule of the liver (blue arrow). T3 represents low penetration of mononuclear inflammatory cells in the triad port (blue arrow), hepatocytes being mostly normal, nucleus having no degenerative changes (green arrow), and mild central vein congestion (red arrow).

All pathological parameters of the liver except hemorrhage under the Gleason's capsule in the T3 group had significant differences with the S group (*P* < 0.01). The content of acidophilic cytoplasm and necrosis of the nucleus in the T2 group had a significant difference with the S groups. The severity of these damages in the liver decreased by increasing the concentration of the extract (Table 4 and Figure 4).

### 3.5. Biochemical Parameters of the Kidney and Its Mercury Content in the Control and Treated Groups

The serum levels of urea, creatinine, and uric acid significantly increased in the S group compared to the control group (Table 5). Dose-dependent effects of alfalfa extract reduced the serum level of urea, creatinine, and uric acid in treated groups compared to the S group. The serum level of sodium had no significant change in all groups. The highest level of potassium was observed in the S group that this level had a significant with T1, T2, and control groups. Another finding of the study was a remarkable accumulation of mercury in the liver and kidney of animals in the S group compared to the C group. Prescribing the extract of alfalfa in different doses reduced the accumulation of mercury in the kidney that its lowest level was observed in the T3 group (Table 5).

### 3.6. Histopathological Evaluation of the Kidney of Animals in the Control and Treated Groups

**Figure 5:**
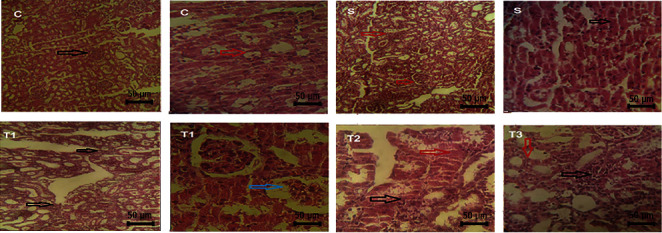
Histopathological changes in the kidney of animals in the control and treated groups (H&E staining, 40x), scale bar = 50 *μ*m. C represents that the glomerular structure was normal (black arrow). There is no bleeding or hyperemia in the glomerulus. Renal tubules are normal (red arrow). Tubular cells are active, and the nucleus of tubular cells are not degenerated. S represents acute tubular damage, acidophilization of cytoplasm (black arrow), destruction of the wall of tubular cells and disintegration of epithelial cells of tubules, and interstitial nephritis (red arrow). T1 represents interstitial nephritis (black arrow), glomerular sclerosis, and increased glomerular space (space between the glomerulus and glomerular wall) (blue arrow). T2 represents extensive penetration of mononuclear inflammatory cells into the interstitial tubular and glomerular space (black arrow) and acute tubular necrosis caused by eosinophilization or acidophilization of the cytoplasm, due to the sedimentation of cytoplasmic proteins (red arrow). T3 represents that mononuclear inflammatory cells were seen in the interstitial space of the tubule (black arrow), the tubules are functional, and no acute tubular necrosis was observed (red arrow).

The acute tubular injury significantly increased in the S and T1 groups compared to the control group. A significant difference between the S group and the control group in the interstitial nephritis was observed, while there was no significant difference with the other groups. The distinctive contrast in glomerular injury was also found in the treatment groups (T1, T2, and T3) and the S group. The hyaline casts in the kidney of the S group and the T1 group significantly increased in comparison to the control group (Table 6; Figure 5).

## 4. Discussion

Mercury is one of the unnecessary heavy metals that does not have any biological role in the body [25]. The body should be free of mercury in physiological conditions but exposed to diet, environmental contact, farm pesticides, and industrial activities. Almost everyone in the world has some mercury in his or her body. Mercury is known to be the third toxic metal after cadmium and lead that humans should avoid [26]. Investigation on oxidative stress indicators is one of the ways to evaluate the chronic damage of mercury to human health [27]. Plants are one of the most important sources of exogenous antioxidants that have important effects on controlling the damage of heavy metals such as mercury [28]. Polyphenols such as phenol and flavonoids are one of the most abundant antioxidant compounds in plants [29]. In this study, alfalfa extract showed a relatively high concentration of phenolic compounds. Our results showed well that reaching IC_50_ at low concentrations shows the high antioxidant activity of alfalfa extract. In this research, body weight was reduced in animals receiving mercury. It was consistent with the findings of other researchers [30, 31]. Oxidative damage from mercury toxicity results in weight loss and muscle cell damage in rats (smooth and striated) [32]. In this study, the administration of alfalfa extract was associated with improving animal weight. It seems that the potential effect of alfalfa by reducing metabolic energy and increasing appetite could improve body weight in rats exposed to mercury. It also inhibits cell damages by inhibiting mercury-induced oxidative stress [33], therefore improving the weight of animals. The results of this study showed that the accumulation of mercury in kidney and liver tissue increased in groups receiving mercury. The concentration of mercury in the blood was variable and unreliable [2]. This could be due to its excellent ability to clear toxins and compounds from the kidney and increase blood flow to the kidney [34]. The kidney is the main target organ of mercury. It causes both glomerular and tubular damage, thus reducing glomerular filtration and renal tubular necrosis [35]. Collecting tubes, especially areas containing the amino acid cysteine, are the site of mercury uptake [36]. Plasma levels of urea, creatinine, and uric acid are the main indicators of kidney function. In this study, the administration of mercury was associated with a significant increase in the level of these indicators, which was consistent with the results of Boroushaki et al. and Mesquita et al. [37, 38]. Therefore, mercury could increase the level of wastewater and waste products by altering the performance of glomerular filtration and the activity of tubules in mercury-induced nephritis [39]. In confirming the nephritis induced by mercury, renal histopathological findings showed acute tubular damage, interstitial nephritis, glomerular damage, and the presence of hyaline cast which was consistent with the results of other researchers. These pathological changes appear to be due to the cell damage induced by oxidative stress [37, 40]. The liver was the second organ considered in this study due to its metabolic role. Liver lysosomes can absorb organic and inorganic mercury and be the site of accumulation of this metal. Mercury binds to glutathione in the liver, blood, and other organs and forms stable sulfhydryl complexes [41]. Nonspecific binding to thiol-SH groups of enzymes can disorder the performance of them, especially antioxidant enzymes, immune responses, protein synthesizers, and energy producers. This can be a reason for the high amount of lipid peroxidation in the kidney and liver due to the increase of mercury concentration. Furthermore, in both kidney and liver organs, the levels of antioxidant enzymes such as GPx, CAT, and SOD decreased when exposed to mercury [42, 43]. This finding shows how the balance of the body's antioxidant system changes after the administration of mercury [44].

ALT and AST are two indicators of the proper and reliable status of liver health. If the liver is damaged, these enzymes leak from the cytosol into the bloodstream, so their concentration increased above normal levels. In this study, these two enzymes showed a significant increase in the group that received mercury. In addition, bleeding, acidophilic cytoplasm, and necrosis of hepatocytes in histological evaluation are the confirmation of this finding [45].

The use of herbal products for the detoxification of heavy metals can be one of the practical and valuable treatment strategies [46]. Alfalfa or green gold has a special place in traditional medicine due to its numerous properties and having protein, calcium, and various vitamins. This plant has the enzymes such as amylase, invertase, and pectinase, which play an important role in digestion and increase in growth. Alfalfa has a high nutritional value. It contains amino acids, namely, Arg, His, Asp, Phe, and Cys, and vitamins, niacin, pantothenic acid, biotin, folic acid, minerals, protein, and saponin [47, 48]. Our results showed that the antioxidant effects of alfalfa extract were in a dose-dependent manner. IC_50_ extract was more than vitamin C that showed the antioxidant importance of the extract compared to vitamin C. Furthermore, in this study, a dose-dependent increase in TAC concentration was observed in the serum of the treated animals with the extract. Moreover, alfalfa contains cysteine that plays an important role in enhancing the detoxification mechanisms of endogenous and heavy metals-induced damage in the body. Moreover, exposure to metals can affect the state of cysteine [49]. Cysteine and its thiol groups can absorb mercury and reduce the damage of thiol groups and proteins in the body's organs, including the liver and kidney [38]. This can be one of the possible reasons for the decrease in mercury concentration in the kidney and liver of the groups treated with alfalfa extract. Alfalfa's nonenzymatic antioxidant compounds (such as phenols and flavonoids) and phytoestrogens, can also be effective in improving liver and kidney enzymatic antioxidants, including glutathione, catalase, and SOD [17]. It could also prevent the accumulation of mercury and the production of free radicals in the liver and kidney in our study.

## 5. Conclusion

The results suggested that mercury chloride could damage the kidney and the liver by inducing oxidative stress. Alfalfa extract by its nutritional and antioxidative activities in a dose-dependent manner could also decrease the toxicity-induced by mercury and could improve the structure and function of the kidney and liver. This extract at a dose of 750 mg/kg showed the highest antioxidant activity against mercury chloride-induced damage in the kidney and liver. Hence, using plant compounds is one of the useful strategies in controlling and preventing heavy metal poisoning.

## Figures and Tables

**Table 1:** Total phenols, total flavonoids, and IC_50_ of the ethanolic extract of alfalfa.

Sample	Total phenolic content (mg GAE/g extract)	Total flavonoid content (mg QE/g extract)	IC_50_ (*μ*g/ml)
Ethanolic extract	40.45 ± 5.19 mg/gr	14.36 ± 1.10	245.18 ± 48.41

**Table 2:** The difference between the basal and final weight of the body in the control and treated groups.

Body weight	Group				
	C	S	T1	T2	T3
Basal	210 ± 15.49	197.5 ± 15.41	205.83 ± 12.81	197.663 ± 7.52	201.66 ± 11.69
Final	237.5 ± 5.24	188.67 ± 11.31 *α*	217.5 ± 16.04 *α*, *β*	212.5 ± 9.54 *α*, *β*	225.65 ± 12.35 *β*

All values are presented as mean ± standard deviation (SD). *P* < 0.001. *α* and *β* denote comparison with C and S groups, respectively.

**Table 3:** Serum levels of liver enzymes and hepatic mercury content in different groups.

Parameter	Group				
	C	S	T1	T2	T3
ALT (U/L)	30.5 ± 3.93	75.5 ± 8.89 *α*	71.33 ± 9.24 *α*	49.83 ± 4.62 *α*, *β*, *γ*	40.00 ± 5.09 *β*, *γ*
AST (U/L)	68.00 ± 5.96	152.66 ± 7.63 *α*	136.67 ± 6.05 *α*, *β*, *γ*	102.67 ± 13.76 *α*, *β*, *γ*	53.00 ± 5.76 *α*, *β*, *γ*
ALP (U/L)	186.66 ± 16.32	275.5 ± 20.03 *α*	256.00 ± 10.19 *α*	236.83 ± 9.68 *α*, *β*	187.65 ± 8.14 *β*, *γ*
Hepatic Hg content (*μ*g/g)	5.18 ± 1.50	15.81 ± 1.68 *α*	13.43 ± 1.06 *α*	11.31 ± 1.30 *α*, *β*	8.05 ± 1.82 *α*, *β*, *γ*

Each row represents the mean ± standard deviation (SD). *P* < 0.001; *α*, *β*, and *γ* denote comparison with C, S, and T1 groups, respectively.

**Table 4:** Histopathological changes in the liver of animals in the control and treated groups.

Parameter	Group				
	C	S	T1	T2	T3
Central vein congestion	0.0 ± 0.3	1.7 ± 0.34 *α*	1.2 ± 0.75 *α*	0.28 ± 1.1 *α*	0.56 ± 0.76 *β*, *γ*
Peripheral hepatitis	0.0 ± 0.1	2.4 ± 1.20 *α*	1.7 ± 1.00 *α*	1.5 ± 0.48 *α*	0.47 ± 0.8 *β*
Hepatocyte necrosis	0.0 ± 0.0	1.27 ± 3.1 *α*	1.6 ± 0.48 *α*, *β*	0.5 ± 0.28 *β*	0.21 ± 0.3 *β*
Parenchymal bleeding	0.0 ± 0.0	0.6 ± 0.09 *α*	0.5 ± 0.02 *α*	0.5 ± 0.06 *α*	0.1 ± 0.05

All values are presented as mean ± standard deviation (SD). *P* < 0.001; *α*, *β*, and *γ* denote comparison with C, S, and T1 groups, respectively.

**Table 5:** The levels of renal parameters in the serum of the control and treated groups.

Parameter	Groups				
	C	S	T1	T2	T3
Urea (mg/dl)	30.41 ± 2.41	46.98 ± 2.59 Α	35.76 ± 2.51 *α*, *β*	37.00 ± 1.41 *α*, *β*	32.90 ± 1.68 *β*
Creatinine (mg/dl)	0.38 ± 0.14	1.23 ± 0.16 *α*, *β*	0.99 ± 0.09 *α*, *β*	0.83 ± 0.05 *α*, *β*	0.75 ± 0.18 *α*, *β*
Uric acid (mg/dl)	1.89 ± 0.14	5.83 ± 1.02 Α	3.6 ± 0.73 *α*, *β*	2.30 ± 0.23 *β*	1.86 ± 0.10 *β*
Sodium (mmol/l)	146.66 ± 6.02	149.83 ± 2.99	142.5 ± 1.64	146.5 ± 4.59	143.5 ± 6.41
Potassium (mmol/l)	4.48 ± 0.91	5.78 ± 0.57 Α	5.45 ± 0.30 *α*	4.83 ± 0.25 *β*	4.26 ± 0.23 *β*
Renal Hg content (*μ*g/g)	5.46 ± 1.59	21.66 ± 3.26 Α	20.00 ± 2.28 *α*	14.05 ± 1.04 *α*, *β*	10.25 ± 1.19 *α*, *β*

All values are presented as mean ± standard deviation (SD). *P* < 0.001; *α* and *β*, respectively, denote comparison with groups C and S.

**Table 6:** Evaluation of the pathological changes in the kidney of the animals in the control and treated groups.

Parameter	Groups				
	C	S	T1	T2	T3
Acute tubular necrosis	0.0 ± 0.0	2.83 ± 0.38 Α	2.66 ± 0.50 *α*	1.33 ± 0.50 *β*, *γ*	0.83 ± 0.74 *β*, *γ*
Interstitial nephritis	0.0 ± 0.0	2.16 ± 0.74 Α	1.33 ± 0.50	1.16 ± 0.38	1.16 ± 0.38
Glomerular damage	0.0 ± 0.0	2.50 ± 0.52 Α	1.0 ± 0.62 *β*	0.83 ± 0.39 *β*	0.56 ± 0.43 *β*
Hyaline cast	0.0 ± 0.0	2.66 ± 0.79 Α	2.50 ± 0.53 *α*	1.16 ± 0.38 *β*	0.83 ± 0.73 *β*

All values are presented as mean ± standard deviation (SD). *P* < 0.01; *α*, *β*, and *γ*, respectively, denote comparison with groups C, S, and T1.

## Data Availability

The data can be obtained upon request to the corresponding author.
